# Upregulated microRNA-125b-5p in patients with asthma-COPD overlap mediates oxidative stress and late apoptosis via targeting IL6R/TRIAP1 signaling

**DOI:** 10.1186/s12931-024-02703-7

**Published:** 2024-02-01

**Authors:** Yu-Ping Chang, Yi-Hsuan Tsai, Yu-Mu Chen, Kuo-Tung Huang, Chiu-Ping Lee, Po-Yuan Hsu, Hung-Chen Chen, Meng-Chih Lin, Yung-Che Chen

**Affiliations:** 1 Division of Pulmonary and Critical Care Medicine, Department of Internal Medicine, Kaohsiung Chang Gung Memorial Hospital and Chang Gung University College of Medicine, No. 123, Dapi Rd., Niaosong Dist., Kaohsiung, 83301 Taiwan (R.O.C.); 2 Department of Respiratory Therapy, Kaohsiung Chang Gung Memorial Hospital and Chang Gung University College of Medicine, No. 123, Dapi Rd., Niaosong Dist., Kaohsiung, 83301 Taiwan (R.O.C.)

**Keywords:** Asthma-COPD overlap, Interleukin 6 receptor, miR-125b-5p, TP53-regulated inhibitor of apoptosis 1

## Abstract

**Background:**

Among patients with chronic obstructive pulmonary disease (COPD), some have features of both asthma and COPD—a condition categorized as asthma-COPD overlap (ACO). Our aim was to determine whether asthma- or COPD-related microRNAs (miRNAs) play a role in the pathogenesis of ACO.

**Methods:**

A total of 22 healthy subjects and 27 patients with ACO were enrolled. We selected 6 miRNAs that were found to correlate with COPD and asthma. The expression of miRNAs and target genes was analyzed using quantitative reverse-transcriptase polymerase chain reaction. Cell apoptosis and intracellular reactive oxygen species production were evaluated using flow cytometry. In vitro human monocytic THP-1 cells and primary normal human bronchial epithelial (NHBE) cells under stimuli with cigarette smoke extract (CSE) or ovalbumin (OVA) allergen or both were used to verify the clinical findings.

**Results:**

We identified the upregulation of miR-125b-5p in patients with ACO and in THP-1 cells stimulated with CSE plus OVA allergen. We selected 16 genes related to the miR-125b-5p pathway and found that *IL6R* and *TRIAP1* were both downregulated in patients with ACO and in THP-1 cells stimulated with CSE plus OVA. The percentage of late apoptotic cells increased in the THP-1 cell culture model when stimulated with CSE plus OVA, and the effect was reversed by transfection with miR-125b-5p small interfering RNA (siRNA). The percentage of reactive oxygen species-producing cells increased in the NHBE cell culture model when stimulated with CSE plus OVA, and the effect was reversed by transfection with miR-125b-5p siRNA. In NHBE cells, siRNA transfection reversed the upregulation of *STAT3* under CSE+OVA stimulation.

**Conclusions:**

Our study revealed that upregulation of miR-125b-5p in patients with ACO mediated late apoptosis in THP-1 cells and oxidative stress in NHBE cells via targeting *IL6R* and *TRIAP1*. *STAT3* expression was also regulated by miR-125b-5p.

## Background

Among patients with chronic obstructive pulmonary disease (COPD), approximately 15–55% exhibit features of both asthma and COPD—a condition categorized as asthma-COPD overlap (ACO) syndrome [1]. Patients with ACO experience more frequent acute exacerbations (AEs), more rapid lung function decline, and higher mortality rate [2]. Systemic inflammation in COPD and asthma is driven by T helper (Th)1 and Th2 immune responses, respectively, while both innate and adaptive immune responses contribute to airway remodeling [3]. However, little is known about innate immune responses, systemic inflammation, and their effects on airway remodeling in ACO.

MicroRNAs (miRNAs) are noncoding endogenous RNAs that are approximately 19 to 25 nucleotides long and participate in posttranscriptional gene regulation [4]. Several miRNAs have been reported to play a role in lung development, immune responses, and lung diseases, such as lung cancer, asthma, COPD, and pulmonary fibrosis [4]. Previous studies have shown that several miRNAs, including miR-21-5p, miR-106b-5p, miR-125b-5p, miR-146a-5p, miR-146b-3p, and miR-223-5p, are either upregulated or downregulated in patients with asthma or COPD, respectively, and have been associated with frequent AEs, high symptom burden, or rapid lung function decline [5–17]. However, few studies have been reported on the role of miRNAs in the pathophysiology of ACO. The aim of this study was to determine whether these asthma- or COPD-related miRNAs play a role in the development and clinical presentations of patients with ACO.

## Material and methods

### Study participants

A total of 22 healthy subjects and 27 patients with ACO were recruited from the health examination center and pulmonary clinic of Kaohsiung Chang Gung Memorial Hospital during the period from May 2013 to July 2017. Written informed consent was obtained from each participant. This study was approved by the Institutional Review Board of Chang Gung Memorial Hospital (IRB: 201800976B0). Data regarding the baseline characteristics of patients and pulmonary function tests were collected during their first visit. Patients were routinely followed-up, and data and pulmonary function tests were collected at least 1 year later or as needed. COPD was diagnosed according to the following criteria: male, age > 40 years, smoking habit ≥ 10 pack/year, and postbronchodilator (BD) forced expiratory volume in one second (FEV_1_)/forced vital capacity (FVC) < 70%. Post-BD improvement in FEV_1_ of > 200 mL and 12% was defined as BD responsive. Patients were excluded if they had a history of lung cancer, tuberculosis, bronchiectasis, or recent admission in the last 4 weeks. COPD AE was defined as worsening of at least 2 of the following 3 symptoms: sputum volume, sputum purulence, and dyspnea. Moderate AE was defined as a condition requiring the prescription of oral antibiotics or systemic steroids. Severe AE was defined as a condition requiring an emergency department stay for more than 24 h or admission.

ACO was defined according to the following criteria: COPD diagnosis with BD responsiveness documented at least once in pulmonary function test, together with either blood eosinophil level > 3% or a history of atopic diseases, such as asthma, allergic rhinitis, or atopic dermatitis. Healthy subjects were defined as never-smokers with normal lung function and without obvious symptoms related to lung disease. Dyspnea score was recorded using the modified Medical Research Council (mMRC) and COPD assessment test (CAT) scores [18, 19]. The lymphocyte to monocyte ratio (LMR) was calculated by dividing the lymphocyte count by the monocyte count. The neutrophil-to-lymphocyte ratio (NLR) was calculated by dividing the neutrophil count by the lymphocyte count.

### Blood collection and RNA extraction from peripheral blood mononuclear cells

For this experiment, 20 mL fresh venous blood was collected from all study subjects after inclusion and immediately transferred to a tube containing 3.2% sodium citrate (1:10 dilution). Peripheral blood mononuclear cells (PBMCs) were isolated using a two-layer Ficoll-Histopaque density gradient centrifugation method (HISTOPAQUE®-119, Sigma-Aldrich, Inc., Burlington, MA, USA). Samples were stored in RNAlater (Ambion Inc., Austin, TX, USA) at − 80 °C until analysis. An miRNeasy Mini Kit column-based system (Qiagen, Valencia, CA, USA) was used for isolation of total RNA, which was treated with DNase according to the manufacturer’s protocol.

### Analysis of expression of microRNAs using quantitative reverse-transcriptase polymerase chain reaction

cDNA was generated from 2 µL of purified total RNA using the TaqMan Advanced miRNA cDNA Synthesis kit (Thermo Fisher Scientific, Waltham, MA, USA). Additionally, 1 pM of the synthetic *Caenorhabditis elegans* oligo, cel-miR-54-3p was added to the isolated total RNA as an exogenous control. All miRNA expression levels were normalized to their corresponding cel-miR-54-3p Ct values. qRT-PCR was performed for each sample using 2.5 µL diluted cDNA, TaqMan Advanced miRNA Assays (cel-miR-54-3p: 001361; hsa-miR-21-5p: 477975_mir; hsa-miR-106b-5p: 478412_mir; hsa-miR-125b-5p: 477885_mir; hsa-miR-146a-5p: 478399_mir; hsa-miR-146b-3p: 483103_mir; and hsa-miR-223-5p: 477984_mir; Thermo Fisher Scientific), and Applied Biosystems™ TaqMan™ Fast Advanced Master Mix (Thermo Fisher Scientific) under fast cycling conditions. All TaqMan qRT-PCR assays were carried out using the ABI 7500fast Real-Time PCR System (Applied Biosystems, Foster City, CA, USA). Real-time PCR cycling conditions consisted of the following steps: 95 °C for 20 s, followed by 40 cycles of 95 °C for 3 s and 60 °C for 30 s. The fold-changes in the expression of all miRNAs were determined using the 2^−∆∆Ct^ method.

### Expression analysis of predicted target genes using qRT-PCR

The expression of predicted target genes was analyzed using qRT-PCR in a 96-well format. The housekeeping gene, *GAPDH*, was chosen as an endogenous control to normalize the expression data of each gene. All PCR primers (random hexamers) were designed and purchased from Roche according to the company’s protocols (www.roche-applied-science.com) (Table 1). RNA samples were treated in a DNA-free manner to remove contaminating genomic DNA. A total of 300 ng RNA was used for the synthesis of first-strand cDNA with the QuantiTectReverse Transcription Kit (QIAGEN, Hilden, Germany), and 5 µL of the master mix (QIAGEN, SYBR Green PCR kit; Roche, Hilden, Germany) was added to each reverse transcription reaction. PCR was performed in a Roche Light CyclerQuantiFast R system for 45 cycles of amplification. A single real-time PCR experiment was carried out for each target gene using each sample. The relative expression was calculated using the 2^−∆∆Ct^ method.

**Table 1:** Sequences of primers used in the quantitative reverse-transcriptase polymerase chain reaction

Gene	Primer sequence (5′ to 3′)	
*BCL2*	Forward	TTGTGGCCTTCTTTGAGTTCGGTG
	Reverse	GGTGCCGGTTCAGGTACTCAGTCA
*CCR2*	Forward	CCACATCTCGTTCTCGGTTTATC
	Reverse	CAGGGAGCACCGTAATCATAATC
*COL4A3*	Forward	ATGGCATCATCGACCTCCCT
	Reverse	GTCACTCGCATGTGTGGGT
*DRAM2*	Forward	CTGTGCTTACCTTTGGTATGGG
	Reverse	GCACTTACTCCACACCAGATAAC
*EDN1*	Forward	AGAGTGTGTCTACTTCTGCCA
	Reverse	CTTCCAAGTCCATACGGAACAA
*FOXP3*	Forward	GTGGCCCGGATGTGAGAAG
	Reverse	GGAGCCCTTGTCGGATGATG
*IFNG*	Forward	TAACTGACTTGAATGTCCAACG
	Reverse	GCAGGCAGGACAACCATTA
*IL10RA*	Forward	CCTCCGTCTGTGTGGTTTGAA
	Reverse	CACTGCGGTAAGGTCATAGGA
*IL6R*	Forward	AGCCTCCCAGTGCAAGATTC
	Reverse	GGTATTGTCAGACCCCAGGC
*IRF4*	Forward	GCGGTGCGCTTTGAACAAG
	Reverse	ACACTTTGTACGGGTCTGAGA
*MSRB3*	Forward	CGGTTCAGGTTGGCCTTCATT
	Reverse	GTGCATCCCATAGGAAAAGTCA
*NLRC5*	Forward	GCTCGGCAACAAGAACCTGT
	Reverse	GGTCCAAGGTCTCGTTCCT
*SOD2*	Forward	GCTCCGGTTTTGGGGTATCTG
	Reverse	GCGTTGATGTGAGGTTCCAG
*STAT3*	Forward	ACCAGCAGTATAGCCGCTTC
	Reverse	GCCACAATCCGGGCAATCT
*TRIAP1*	Forward	CGAGTACGACCAGTGCTTCA
	Reverse	CTCTTTCTCCTTTATTGCTTTCTGA
*VDR*	Forward	TCTCCAATCTGGATCTGAGTGAA
	Reverse	GGATGCTGTAACTGACCAGGT

### In vitro human monocytic THP-1 cells and primary normal human bronchial epithelial cells under stimuli with cigarette smoke extract or ovalbumin allergen or both

The human monocytic cell line THP-1 (ATCC® TIB-202™) was purchased from American Type Culture Collection (ATCC) (Manassas, VA, USA). THP-1 cells were cultured in RPMI 1640 medium (Thermo Fisher Scientific) containing 10% fetal bovine serum (FBS), 100 U/mL penicillin, and 100 μg/mL streptomycin. Davidoff cigarettes containing 10 mg tar and 0.8 mg nicotine per cigarette were used for preparing the cigarette smoke extract (CSE) according to previous methods [20, 21] with modifications. THP-1 cells were treated with normal medium, 2.5% CSE, 25 μg ovalbumin (OVA), or CSE (2.5%) plus OVA (25 μg) for 48 h.

Primary normal human bronchial epithelial (NHBE) cells (ATCC® PCS-300-010™) obtained from ATCC were cultured in ATCC Airway Epithelial Cell Basal Medium (ATCC® PCS-300-030™) supplemented with Bronchial Epithelial Cell Growth Kit (ATCC® PCS-300-040™). NHBE cells were treated with CSE or OVA or both in the same manner as THP-1 cells.

Gene expression was measured using qRT-PCR with a Taqman probe (Thermo Fisher Scientific) and specific primers. The relative expression was calculated using the 2^−∆∆Ct^ method.

### Evaluation of cell apoptosis using flow cytometry

After treatment, cells were washed twice with phosphate-buffered saline (PBS) (Thermo Fisher Scientific), resuspended in binding buffer (Thermo Fisher Scientific), and incubated with 5 µL FITC-Annexin V and 5 µL propidium iodide (PI) for 15 min at 25 °C. The cells were evaluated using an Annexin V/propidium iodide apoptosis detection kit (BD Biosciences, Franklin Lakes, NJ, USA) in a FACScan flow cytometry system (Becton Dickinson, San Diego, CA, USA).

### Evaluation of intracellular reactive oxygen species

A fresh stock of 0.1 µM solution of H2DCFDA (catalog no. D6883; Sigma Aldrich) was added to cells seeded at a density of 1 × 10^6^ cells/mL. The cell-associated mean fluorescent intensity was measured using flow cytometry in the FL1 channel at excitation and emission wavelengths of 488 and 535 nm, respectively, using the Cytomics™ FC500 (Beckman Coulter, Brea, CA, USA).

### Evaluation of luciferase activity

The pmirGLO Dual-Luciferase miRNA Target Expression Vector (pmirGLO) (Promega, Madison, WI, USA) was used for the luciferase reporter assay. Briefly, two plasmid constructs, the *IL6R* wild type and *IL6R* mutated type, were created and cotransfected in cells using the Lipofectamine 3000 reagent (Thermo Fisher Scientific) along with different concentrations of the miR-125b-5p mimic (0, 5, 10, 25, and 50 µM) using the HiPerFect transfection reagent (Qiagen). The Dual-Glo® Luciferase Assay System (Promega) was used to measure the luciferase activity.

### Transfection with miR-125b-5p small interfering RNA

The miR-125b-5p small interfering RNA (siRNA) (concentration, 5/10/25 µM) was synthesized by GenePharma and was transfected in cells using the Lipofectamine 2000 reagent (Invitrogen, Carlsbad, CA, USA). The HiPerFect transfection reagent (Qiagen) was added to the culture for 24 h to induce the overexpression of miR-125b-5p siRNA. The knockdown efficiency was detected using qRT-PCR.

### Statistical analysis

Statistical analyses were performed using the SPSS version 21.0 (IBM Corp., Armonk, New York, USA). Categorical variables were compared using the Chi-square test and presented as frequency with percentage. Normally distributed continuous variables were presented as the mean and standard deviation and compared using the independent sample t test or one-way analysis of variance. Continuous variables not normally distributed were presented as the median with interquartile range and compared using the nonparametric Mann–Whitney *U* test or Kruskal–Wallis test. Fold change values were calculated using the 2^−∆∆Ct^ method. Statistical significance was set at a *p*-value of < 0.05.

## Results

### Patient characteristics

We enrolled a total of 22 healthy subjects and 27 patients with ACO. The baseline characteristics of all participants are listed in Table 2. We did not detect any significant differences in age, body mass index, age-adjusted Charlson comorbidity index, percentage of blood neutrophil, lymphocyte and monocyte, NLR, LMR, hemoglobin, or albumin. However, we observed that the white blood cell counts of healthy subjects were lower than those in patients with ACO. In addition, patients with ACO had a higher percentage of blood eosinophils and absolute eosinophil counts than healthy subjects. Overall, healthy subjects had better lung function.

**Table 2:** Comparison of clinical characteristics at baseline between healthy subjects and patients with ACO

	Healthy subjects (*n* = 22)	Patients with ACO (*n* = 27)	*p*-value
Age (years)	67.5 (9.5)	67.3 (11.4)	0.947
BMI (kg/m^2^)	25.0 (4.0)	23.1 (5.1)	0.152
Current smoker (%)	0 (0%)	8 (29.6%)	0.006
Pack/year	0	50.0 (30.0–75.0)	< 0.001
ACCI	4.0 (2.4)	4.7 (1.9)	0.230
mMRC score	N.A.	2.0 (1.0–3.0)	
CAT score	N.A.	9.0 (6.0–15.0)	
Hemogram parameters			
WBC count (1000/μL)	5.9 (2.4)	8.0 (2.5)	0.004
Neutrophil (%)	59.9 (9.4)	56.4 (13.9)	0.324
Lymphocyte (%)	31.2 (10.6)	32.0 (12.0)	0.806
Monocyte (%)	6.5 (2.2)	6.4 (1.9)	0.928
Eosinophil (%)	1.8 (0.8–2.9)	3.2 (1.9–5.1)	0.008
AEC (/μL)	90.4 (48.6–158.2)	291.2 (121.8–432.0)	0.001
NLR	1.9 (1.3–2.6)	1.9 (1.1–2.6)	0.469
LMR	6.2 (3.5–7.5)	4.6 (3.7–6.9)	0.644
Hemoglobin (g/dL)	13.7 (1.8)	14.2 (1.6)	0.271
Albumin (g/dL)	4.2 (0.4)	4.4 (0.4)	0.247
Pulmonary function test			
Pre-BD FVC (%)	91.8 (10.9)	74.0 (16.8)	< 0.001
Pre-BD FEV_1_ (%)	93.9 (10.9)	49.9 (17.3)	< 0.001
Pre-BD FEV_1_/FVC	78.9 (75.8–84.1)	52.9 (45.1–60.3)	< 0.001
Pre-BD FEF_25%–75%_ (%)	82.0 (31.1)	20.2 (9.3)	< 0.001
Post-BD FVC (%)	N.A.	77.4 (16.4)	
Post-BD FEV_1_ (%)	N.A.	54.7 (18.9)	
Post-BD FEV_1_/FVC	N.A.	53.7 (11.3)	
Post-BD FEF_25%–75%_ (%)	N.A.	23.8 (11.9)	
BD response (%)	N.A.	2 (7.4%)^a^	
Atopic diseases			
Asthma history	0 (0%)	14 (51.9%)	< 0.001
Allergic rhinitis	0 (0%)	14 (51.9%)	< 0.001
Atopic dermatitis	0 (0%)	3 (11.1%)	0.242
AEs in the previous year	N.A.	2.0 (1.0–4.0)	

^a^Bronchodilator response at enrollment, but 100% with documented bronchodilator response in other pulmonary function tests

### Differential expression of the six selected microRNAs in the patient cohort and in the human monocytic THP-1 cell culture model

**Fig. 1:**
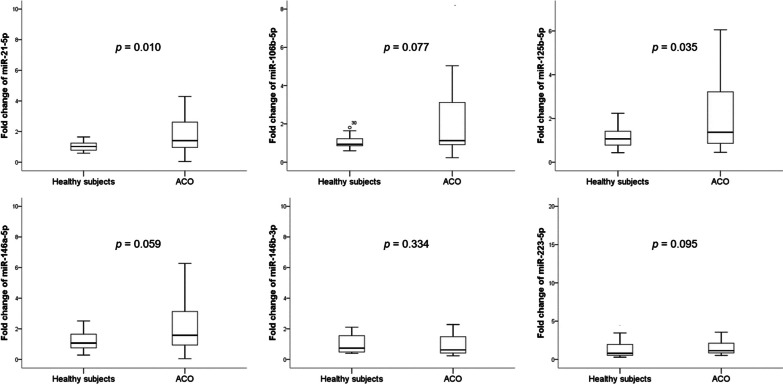
Relative expression levels of microRNAs in the patient cohort. Fold change values of microRNAs in the patient cohort. *p < 0.05; **p < 0.001

We searched PubMed online for miRNAs related to both asthma and COPD, and identified 6 miRNAs: miR-21-5p [5–7], miR-106b-5p [7, 8], miR-125b-5p [9–11], miR-146a-5p [7, 12–14], miR-146b-3p [13, 15], and miR-223-5p [7, 16, 17]. Thus, we evaluated the levels of expression of these 6 selected miRNAs using qRT-PCR. We found that 2 miRNAs, miR-21-5p and miR-125b-5p, showed significant differential expression in the patient cohort (Fig. 1). Both miR-21-5p and miR-125b-5p were upregulated in patients with ACO compared with that in healthy subjects.

**Fig. 2:**
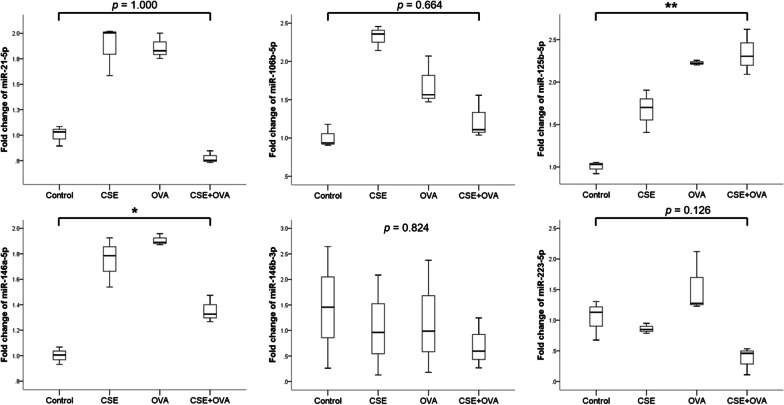
Relative expression levels of microRNAs in the THP-1 cell culture model. Fold change values of microRNAs in the THP-1 cell culture model (n = 3). *p < 0.05; **p < 0.001

We then performed an in vitro human monocytic THP-1 cell culture under stimulation with CSE or OVA allergen or both to test whether the expression patterns of these miRNAs in vitro would correspond with those from the blood samples of patients. We observed that miR-21-5p, miR-106b-5p, miR-125b-5p, miR-146a-5p, and miR-223-5p showed differential expression (Fig. 2). However, only miR-125b-5p and miR-146a-5p were upregulated in CSE+OVA costimulated THP-1 cells compared with those in control. We selected miR-125b-5p for further evaluation because its level of expression was increased both in THP-1 cells stimulated with CSE+OVA and patients with ACO.

### Levels of expression of predicted target genes of miR-125b-5p in the patient cohort and in the THP-1 cell culture model

**Fig. 3:**
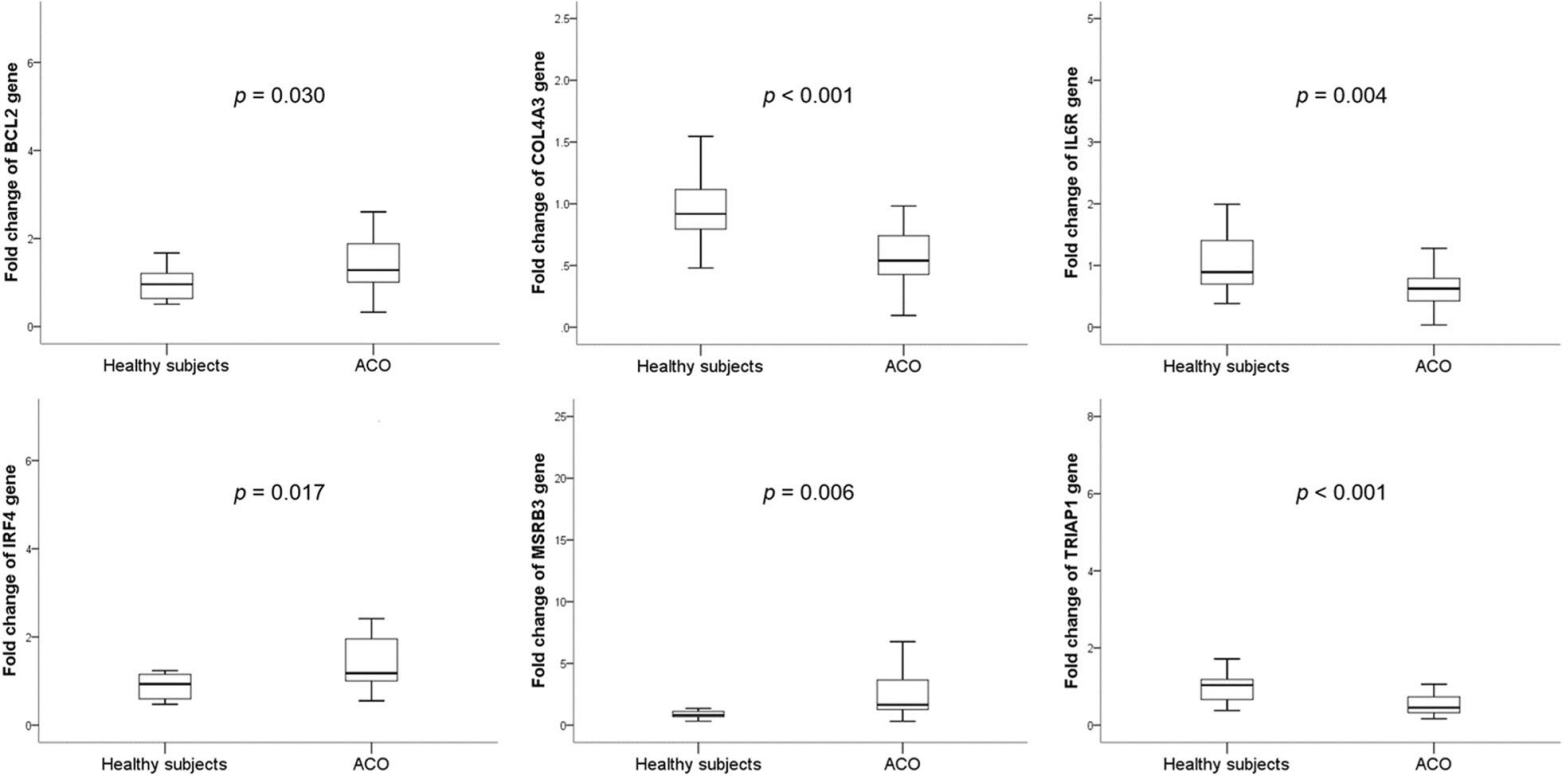
Relative expression levels of target genes in the patient cohort. Fold change values of target genes in the patient cohort. *p < 0.05; **p < 0.001

We searched the PubMed database, miRDB (http://mirdb.org/cgi-bin/search.cgi?searchType=miRNA&full=mirbase&searchBox=MIMAT0000423) and TargetScan (V.8.0; TargetScanHuman 8.0) online for predicting target genes related to the function of miR-125b-5p. We identified a total of 16 target genes, namely *BCL2*, *CCR2*, *COL4A*, *DRAM2*, *EDN1*, *FOXP3*, *IFNG*, *IL10RA*, *IL6R*, *IRF4*, *MSRB3*, *NLRC5*, *SOD2*, *STAT3*, *TRIAP1*, and *VDR*. We evaluated the levels of expression of these genes in the same PBMC samples using qRT-PCR. We found that 6 genes, *BCL2*, *COL4A3*, *IL6R*, *IRF4*, *MSRB3*, and *TRIAP1*, showed significant differential expression in the patient cohort (Fig. 3). Among them, *COL4A3*, *IL6R*, and *TRIAP1* were downregulated in patients with ACO, whereas *BCL2*, *IRF4*, and *MSRB3* were upregulated in patients with ACO compared with that in healthy subjects.

**Fig. 4:**
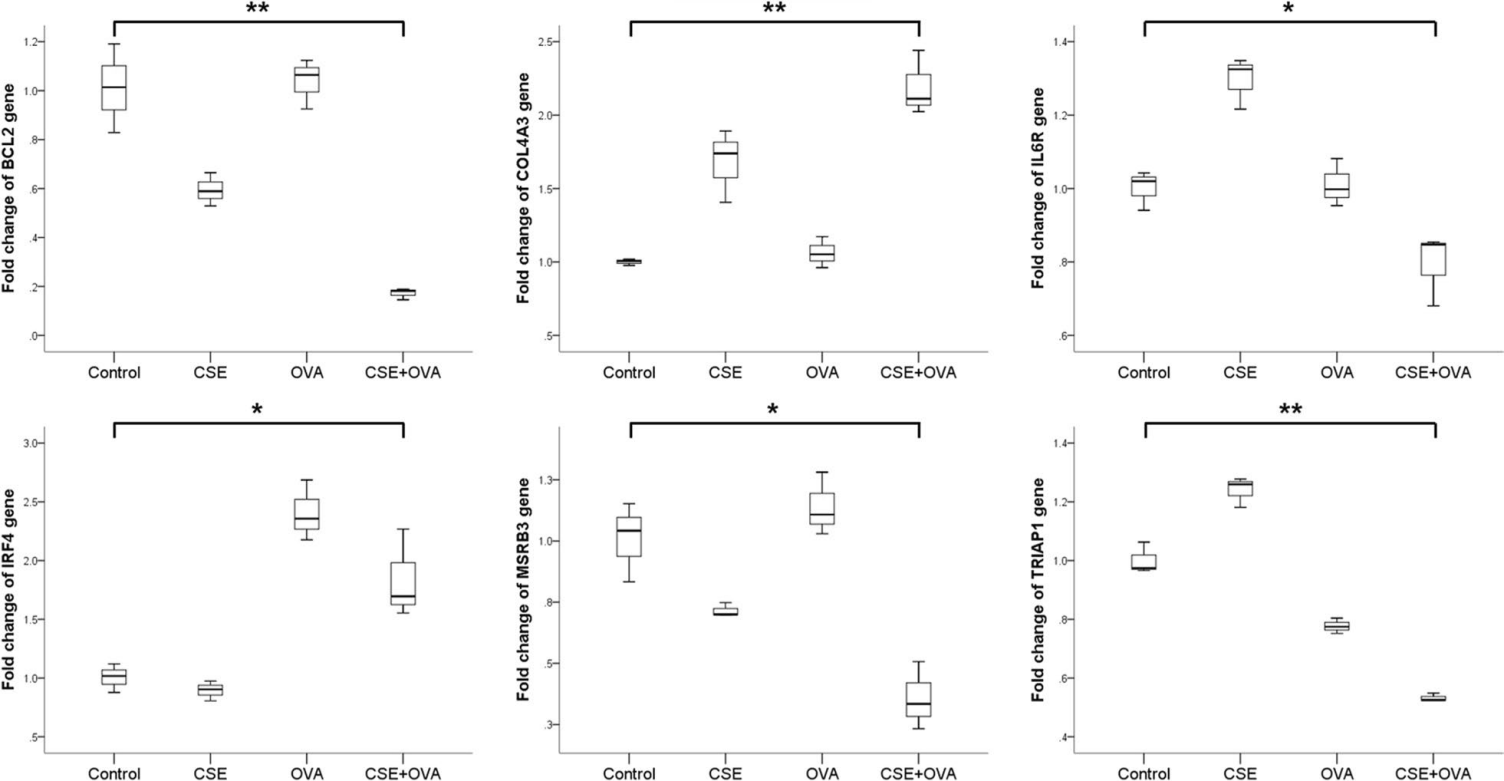
Relative expression levels of target genes in the THP-1 cell culture model. Fold change values of target genes in the THP-1 cell culture model (n = 3). *p < 0.05; **p < 0.001

We also noticed that the expression levels of these 6 predicted target genes were all significantly changed in human monocytic THP-1 cells stimulated with CSE or OVA or both (Fig. 4). *COL4A3* and *IRF4* were upregulated in the CSE+OVA group compared with that in the control group. *BCL2*, *IL6R*, *MSRB3*, and *TRIAP1* were downregulated in the CSE+OVA group compared with that in the control group. Thus, we found that *IL6R* and *TRIAP1* were consistently downregulated both in patients with ACO and in response to CSE+OVA costimulus.

### Knockdown of miR-125b-5p reversed CSE+OVA-induced late apoptosis in THP-1 cells

**Fig. 5:**
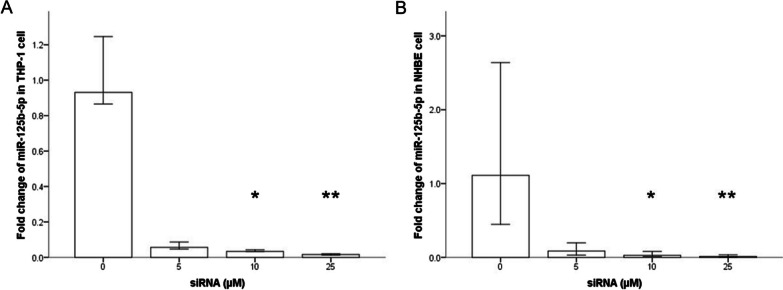
Knockdown efficiency of miR-125b-5p siRNA. Knockdown efficiency of miR-125b-5p siRNA in the THP-1 (n = 6) (**A**) and NHBE (n = 6) (**B**) cell culture models. *p < 0.05 compared with the control group; **p < 0.001 compared with the control group

To determine the detrimental effect of miR-125b-5p, we evaluated cell apoptosis and intracellular production of ROS in THP-1 cells stimulated with CSE or OVA or both. We transfected cells with miR-125b-5p siRNA at a concentration of 10 µM; the knockdown efficiency is shown in Fig. 5A.

**Fig. 6:**
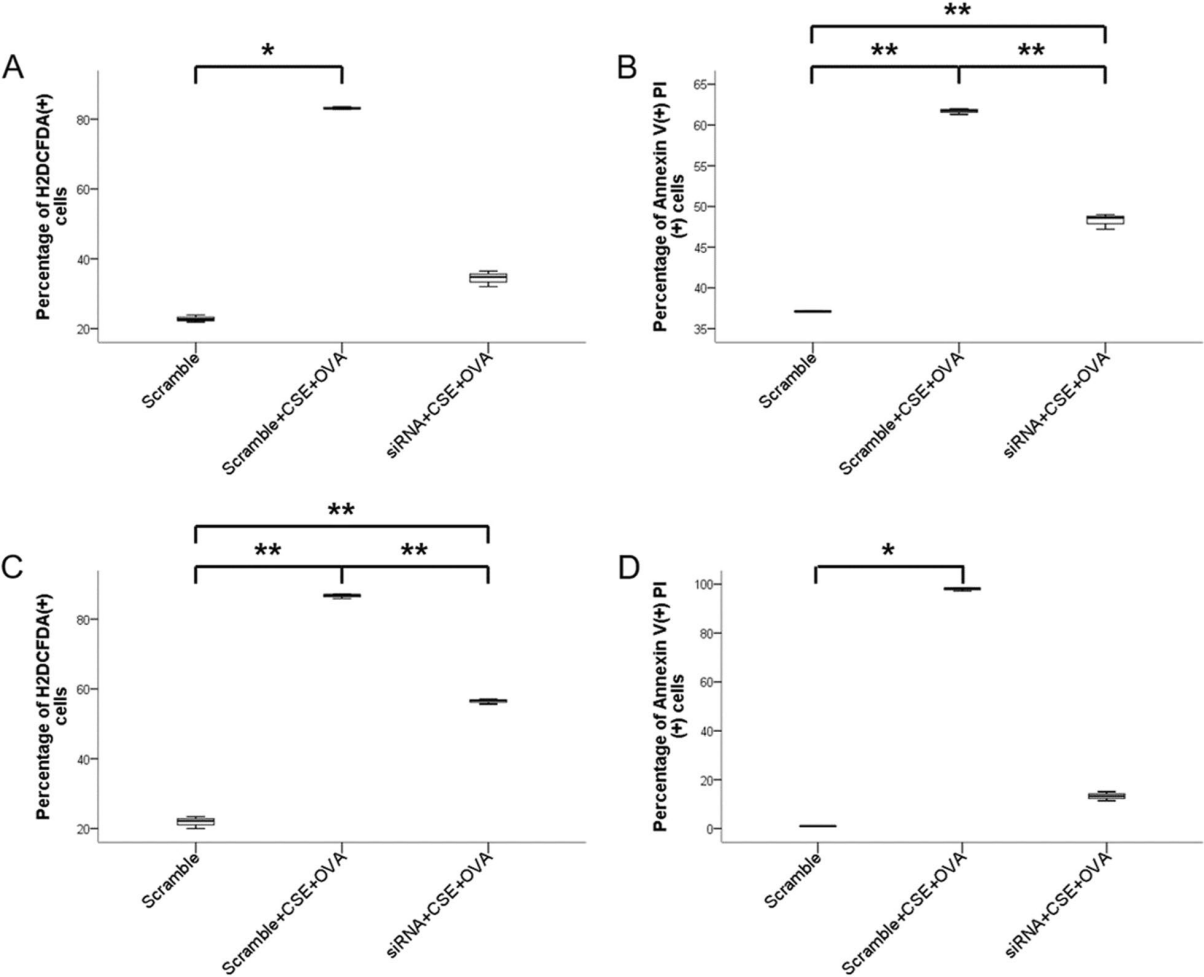
The effect of CSE+OVA and miR-125b-5p siRNA in mediating oxidative stress and late apoptosis. Percentage of reactive oxygen species (ROS)-producing cells (n = 3) (**A**) and late apoptotic cells (n = 3) (**B**) in the THP-1 cell culture model. Percentage of ROS-producing cells (n = 3) (**C**) and late apoptotic cells (n = 3) (**D**) in the NHBE cell culture model. In both the THP-1 and NHBE cell culture models, the cells were stimulated with CSE and OVA before and after transfection with 10 µM miR-125b-5p siRNA. *p < 0.05; **p < 0.001

**Fig. 7:**
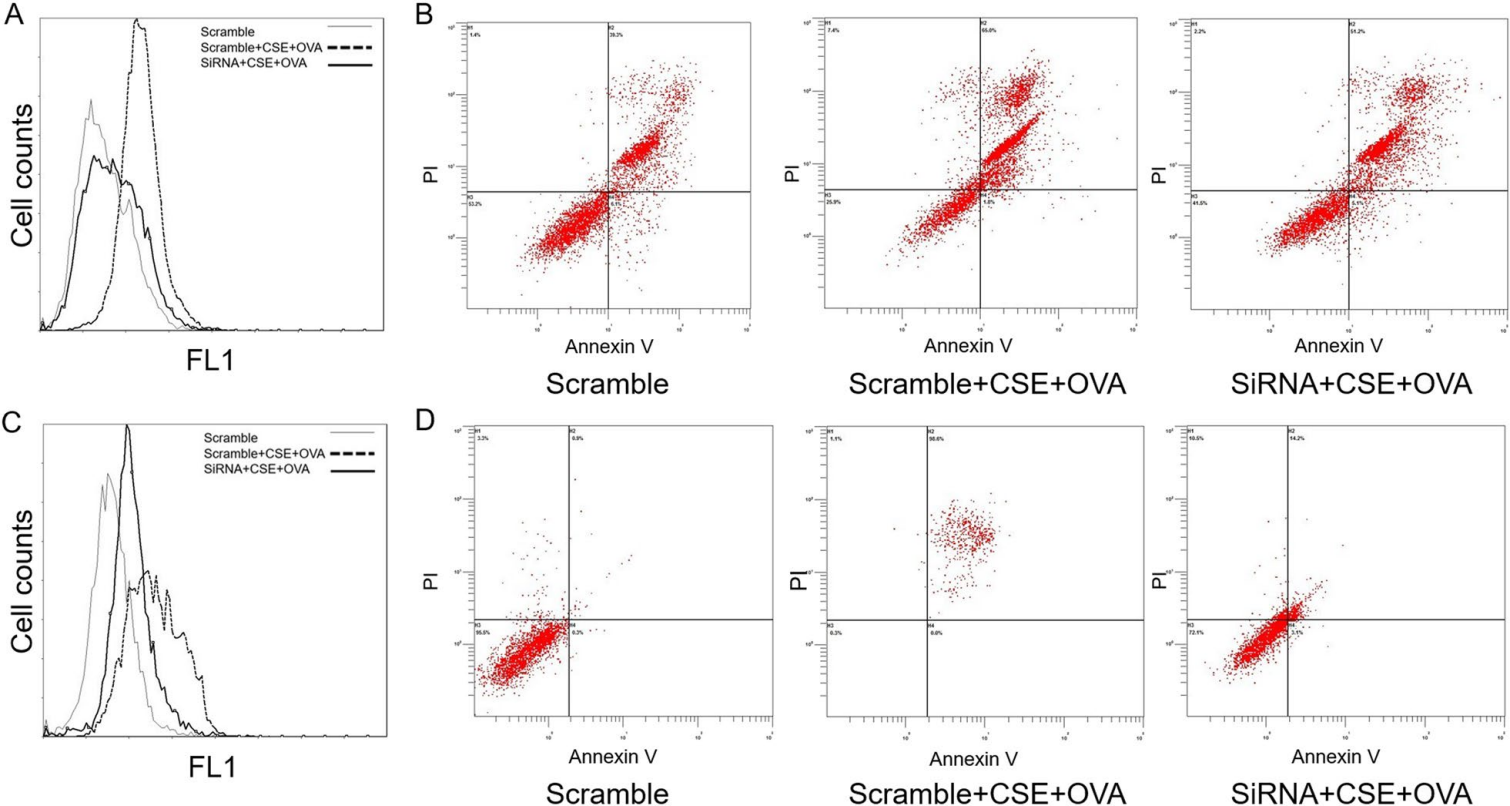
Representative flow cytometry images of intracellular ROS levels and cell apoptosis. Representative flow cytometry curves of total ROS levels in the THP-1 cell (**A**) and NHBE cell (**C**) culture models. Representative flow cytometry images of cell apoptosis in the THP-1 cell (**B**) and NHBE cell (**D**) culture models

We found that in THP-1 cells costimulated with CSE and OVA, both the percentage of ROS-overproducing cells and late apoptotic cells increased significantly compared with that in the scramble group, but after transfection with 10 µM siRNA, only the percentage of late apoptotic cells decreased significantly (Fig. 6A, B). Representative flow cytometry images are shown in Fig. 7A, B.

### Knockdown of miR-125b-5p reversed CSE+OVA-stimulated oxidative stress in normal human bronchial epithelial cells

The knockdown efficiency of miR-125b-5p siRNA in the NHBE cell culture model is shown in Fig. 5B. We observed that oxidative stress and late apoptosis increased in NHBE cells when costimulated with CSE and OVA compared with those in the scramble group, but only the effect on oxidative stress decreased significantly after transfection with 10 µM miR-125b-5p siRNA (Fig. 6C, D). Representative flow cytometry images are shown in Fig. 7C, D.

### Knockdown of miR-125b-5p reversed the effect of CSE+OVA co-stimulation on the expression levels *IL6R* and *STAT3* in the THP-1 and NHBE cell culture models

**Fig. 8:**
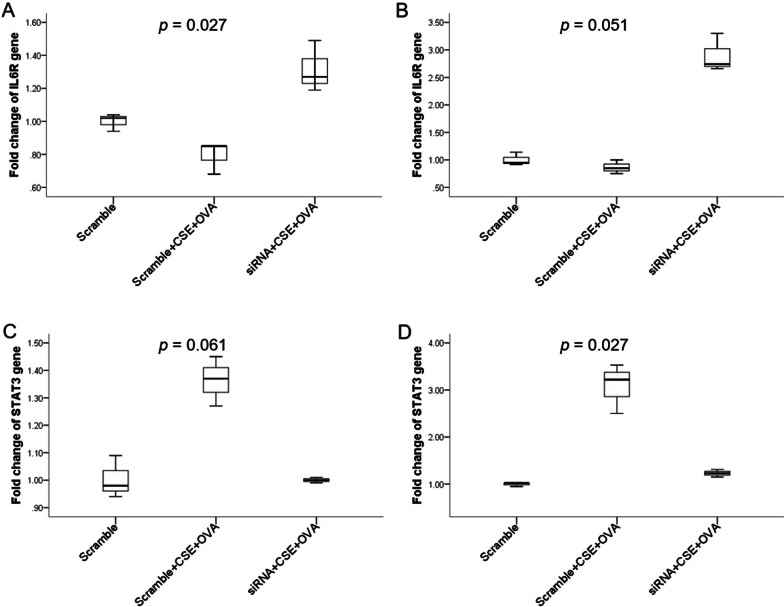
The expression levels of *IL6R* and *STAT3* under co-stimulation with CSE+OVA and after transfection with miR-125b-5p siRNA in the THP-1 and NHBE cell culture models. The expression levels of *IL6R* in the THP-1 (n = 3) (**A**) and NHBE (n = 3) (**B**) cell culture models. The expression levels of *STAT3* in the THP-1 (n = 3) (**C**) and NHBE (n = 3) (**D**) cell culture models. In both the THP-1 and NHBE cell culture models, the cells were stimulated with CSE and OVA before and after transfection with 10 µM miR-125b-5p siRNA

The expression levels of *IL6R* were downregulated after co-stimulation with CSE+OVA in both the THP-1 and NHBE cell culture models (Fig. 8A, B). This effect was reversed after transfection with miR-125b-5p siRNA. But the differential expression only reached statistical significance in the THP-1 cell group, not in the NHBE cell group. The expression levels of *STAT3* were upregulated when stimulated with CSE+OVA and downregulated after transfection with miR-125b-5p siRNA in both the THP-1 and NHBE cell culture models (Fig. 8C, D). The differential expression is significant in the NHBE cell group but not in the THP-1 cell group.

### miR-125b-5p directly bound to IL6R mRNA

**Fig. 9:**
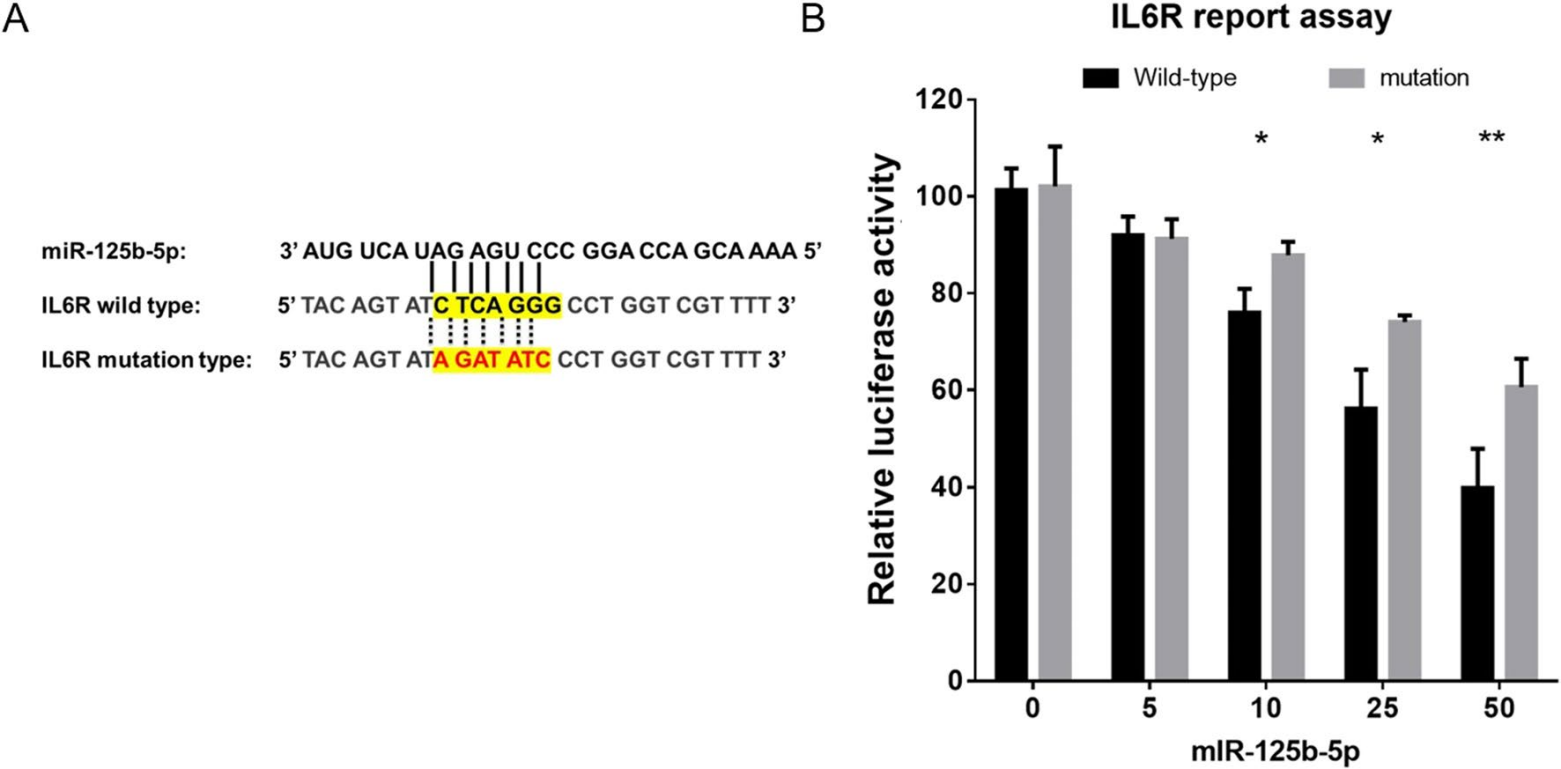
Luciferase reporter assay for miR-125b-5p binding sites in the 3′-untranslated region of *IL6R*. **A** Wild-type and mutated putative miR-125b-5p binding sites in the 3′-untranslated region of *IL6R*. **B** Direct binding activity between miR-125b-5p and *IL6R* assessed using luciferase reporter assays. *p < 0.05; **p < 0.01

We suspected that miR-125b-5p was involved in the expression of *IL6R* and therefore conducted a dual-luciferase reporter assay to test whether miR-125b-5p binds directly to IL6R. To examine whether miR-125b-5p could directly target *IL6R* mRNA, we constructed a luciferase reporter carrying a wild or mutated putative binding site of miR-125b-5p in the 3′-untranslated region of *IL6R* and transfected it into THP-1 cells. As shown in Fig. 9, the miR-125b-5p mimic dose-dependently inhibited the luciferase activity of the wild-type IL6R reporter but displayed no obvious effects on the activity of the mutant IL6R reporter, suggesting the target-binding site of IL6R for miR-125b-5p.

## Discussion

In the current study, we deciphered the role of miR-125b-5p in causing overproduction of oxidative stress and late apoptosis via targeting IL6R/TRIAP1 signaling in patients with ACO.

### Role of miR-125b-5p in COPD, asthma, and ACO pathogenesis

Several researchers have tried to explore the role of miR-125b-5p in patients with COPD or asthma. Hu et al. showed that serum miR-125b correlated with COPD AE and was also positively correlated with the expression of tumor necrosis factor-alpha (TNF-α), interleukin-8 (IL-8), and leukotriene B4 [9]. In addition, the serum levels of miR-125b were found to be increased in patients with asthma and allergic rhinitis [11]. miR-125b-5p was also upregulated in lung tissues in a mouse model treated with long-term ovalbumin [22]. Other studies have reported controversial results, such as the reduced expression of miR-125b and let-7c in the sputum of patients with COPD [10] and the downregulation of miR-125b-1 in lung tissues of patients with COPD compared with that in smokers without COPD [23]. In Roffel’s study, they detected a lower expression of miR-125b-5p in bronchial biopsy specimens from severe asthmatic patients, and the expression of miR-125b-5p was positively associated with FEV_1_ (%) but negatively associated with blood neutrophil counts [24]. The expression pattern of miR-125b-5p was inconsistent in patients with COPD and asthma according to these studies, suggesting that miR-125b-5p might participate in several aspects of COPD and asthma.

Macrophages were dysregulated in COPD. M1 macrophages, which are cytotoxic and proinflammatory, participate in Th1 cytokine-related immune reactions, whereas M2 macrophages, which are anti-inflammatory and linked with tissue repair and fibrosis, participate in Th2 cytokine-related immune reactions [25]. miR-125b-5p, along with miR-125a-5p, miR-181a-5p, and miR-193b-3p, was reported to be significantly upregulated in interferon-gamma (IFN-γ)- and TNF-α-stimulated M1 macrophages and in M2 polarized cells (IL-4-stimulated M2a and IL-10-stimulated M2c) from healthy subjects [26]. Alveolar macrophages in healthy smokers and smokers with COPD showed a predilection toward M2 polarization and M1 deactivation [27]. Another study showed that alveolar macrophages were mostly nonpolarized in normal lungs, whereas dual polarization of M1/M2 was increased with smoking and COPD severity [28]. Thus, miR-125b-5p might participate in both M1 and M2 macrophage polarization pathways in patients with COPD. Rossi reported that naive CD4+ T cells activated by ectopic miR-125b exhibited reduced production of IFN-γ and interleukin-13 (IL-13), indicating a reduced effector function of T lymphocytes [29]; this suggested that miR-125b-5p might inhibit the Th1 differentiation of T lymphocytes, and combined with the miR-125b-5p-induced upregulation in M2 macrophages [26], it could partially explain the miR-125b-5p-induced upregulation in ACO as asthma is mainly characterized by the Th2 differentiation pathway.

### Role of IL6R in COPD, asthma, and ACO pathogenesis

The IL-6 binding receptor (IL-6R) is present in the membrane of some cells, including some leukocytes, where it can be cleaved to produce soluble IL-6R (sIL-6R), which has comparable affinity to interleukin-6 (IL-6) as that of membrane-bound IL-6R [30]. Cell stimulation by the sIL-6R/IL-6 complex with gp130 is called trans-signaling, with more cells being stimulated by trans-signaling due to the universal expression of gp130 [30]. Classic IL-6 signaling through membrane-bound IL-6R has been associated with regenerative activities, whereas trans-signaling has been related to proinflammatory activities and blocking trans-signaling in an emphysema mouse model, where it suppressed alveolar cell apoptosis [30]. IL-6 cytokine promotes the proliferation and reactivation of mast cells and has been found to be related to asthma [31]. In addition, one meta-analysis showed higher serum levels of IL-6 in patients with stable COPD compared with those in healthy controls [32]. sIL-6R was upregulated in the sputum of patients with COPD [33], and cigarette smoke was reported to induce IL-6R shedding in human primary bronchial epithelial cells, especially from patients with COPD [34]. However, in our study, IL6R was downregulated in patients with ACO. One reason is that we used monocyte samples, and downregulation of IL6R in monocytes might decrease classic signaling and increase trans-signaling of the IL-6/sIL-6R pathway, which is associated with proinflammatory activities [30]. Further supporting evidence came from the study by Schmit using an allergic asthma murine model, in which IL-6-deficient mice were characterized by increased infiltration of immune cells, fewer goblet cells, more subepithelial fibrosis around large and distal airways, and an increased number of lung eosinophils compared with that in wild-type asthmatic mice, whereas they did not exhibit any differences in the serum levels of IgE or B- and T-cell frequencies in the lung [35]. Taken together, downregulation of the IL-6 pathway might lead to immune cell infiltration and subepithelial fibrosis. As such, the downregulation of IL6R leading to decreased activity of the IL-6/IL-6R pathway could explain our results.

In addition to *IL6R*, we selected *STAT3* for evaluation because IL-6/signal transducer and activator of transcription 3 (STAT3)-mediated signaling involved in various pathophysiological conditions including asthma and COPD [36, 37]. In our study, *IL6R* gene was downregulated in THP-1 cells upon stimulation with both CSE and OVA, and the effect was reversed by transfection with miR-125b-5p siRNA. The expression levels of *STAT3* were found to be upregulated in NHBE cells when stimulated with both CSE and OVA. This finding is consistent with previous studies regarding the role of IL-6/STAT3 signaling pathway in the pathogenesis of asthma and COPD [38, 39]. Because the effect of miRNA-mediated gene silencing [40], it was anticipated that *STAT3* would be upregulated after miR-125b-5p knockdown. In our study, *STAT3* was downregulated after miR-125b-5p knockdown. This finding suggests that miR-125b-5p may regulate *STAT3* through alternative pathophysiological pathways, rather than directly binding to *STAT3*.

### Role of TRIAP1 in COPD, asthma, and ACO pathogenesis

TP53-regulated inhibitor of apoptosis 1 (TRIAP1) is known to inhibit the apoptotic pathway through interaction with HSP70 and might serve as a marker of drug resistance in breast cancer [41]. TRIAP1 binds directly to miR-125b-5p, and upregulation of miR-125b-5p and downregulation of TRIAP1 were found in both human lumbar degenerative nucleus pulposus (NP) cells and IL-1β-treated NP cells. Interestingly, transfection with a miR-125b-5p inhibitor in IL-1β-treated NP cells prevented apoptosis [42].

In our study, we found that TRIAP1 was downregulated and knockdown with miR-125b-5p siRNA reversed this effect and reduced late apoptosis and ROS overproduction stimulated by CSE and OVA. Thus, the downregulation of TRIAP1 in patients with ACO observed in our study might contribute to ACO pathogenesis through enhancing the apoptotic pathway.

### Ovalbumin versus house dust mite sensitization

OVA and house dust mite (HDM) allergens were used to simulate asthma in animal models [43, 44]. However, the asthma models triggered by OVA or HDM allergens maybe different. In a mouse model study, the pattern of cells in bronchoalveolar lavage fluid (BALF) after acute and chronic exposure to OVA or HDM was different [43]. In another murine model study, HDM triggered more eosinophilia, higher concentrations of IL-4, IL-10, and IFN-γ in BALF compared to OVA, and the author concluded that murine models triggered by HDM were more relevant to human allergic asthma [44]. Therefore, in our study, we used the OVA allergen for stimulation, which may exhibit a different pathophysiological pattern of asthma compared to HDM. Further studies using HDM allergens maybe needed to clarify the role of miR-125b-5p in the pathophysiology of ACO.

### THP-1 cell line versus peripheral blood mononuclear cells

The THP-1 cell line was established from the peripheral blood of a patient with acute monocytic leukemia [45]. The THP-1 cell line has several advantages over PBMCs, such as a higher-growing rate, immortality, and being easier to prepare, and it is widely used to study the pathophysiology of monocytes and macrophages [46]. But the response of THP-1 cells and PBMCs to stimulation may differ. For example, the pattern of cytokine release after lipopolysaccharide stimulation is different between THP-1 cells and PBMCs [47]. Thus, further studies comparing the responses between THP-1 cells and PBMCs are needed to explore the pathways involved in ACO.

### Limitations

Our study had several limitations. First, the patient cohort was small. Second, BD response was not 100% in patients with ACO at enrollment, although all pulmonary function tests of patients with ACO showed a BD response in one of the previous, current, or follow-up tests. Finally, we didn’t collect sputum and bronchial epithelial cells from patients, so we can’t compare the miRNA expression profile with that of PBMC.

## Conclusion

Our study revealed that upregulation of miR-125b-5p in patients with ACO mediated oxidative stress in NHBE cells and late apoptosis in THP-1 cells via targeting *IL6R* and *TRIAP1*. *STAT3* expression was regulated by miR-125b-5p through alternative pathway. Hence, miR-125b-5p siRNA might serve as a novel treatment option. Further studies are required to understand the relationship between the upregulation of miR-125b-5p and long-term outcomes in patients with ACO and to clarify the underlying mechanisms by which knocking down miR-125b-5p leads to less oxidative stress and reduced late apoptosis induced by costimulation with CSE and OVA.

## Data Availability

The data supporting the results in this study are available on request from the first author.

## Abbreviations

ACCI: Age-adjusted Charlson comorbidity index
ACO: Asthma-COPD overlap
AE: Acute exacerbation
AEC: Absolute eosinophil count
BALF: Bronchoalveolar lavage fluid
BD: Bronchodilator
BMI: Body mass index
CAT: COPD assessment test
COPD: Chronic obstructive pulmonary disease
CSE: Cigarette smoke extract
FEF_25%–75%_: Forced expiratory flow between 25 and 75% of vital capacity
FEV_1_: Forced expiratory volume in 1 s
FVC: Forced vital capacity
IFN: Interferon
IL: Interleukin
LMR: Lymphocyte-to-monocyte ratio
miRNA: MicroRNA
mMRC: Modified Medical Research Council
N.A.: Not applicable
NHBE: Normal human bronchial epithelial cells
NLR: Neutrophil-to-lymphocyte ratio
NP: Nucleus pulposus
OVA: Ovalbumin
PBMC: Peripheral blood mononuclear cell
PI: Propidium iodide
qRT-PCR: Quantitative reverse-transcriptase polymerase chain reaction
ROS: Reactive oxygen species
siRNA: Small interfering RNA
STAT3: Signal transducer and activator of transcription 3
Th: T helper
TNF: Tumor necrosis factor
TRIAP1: TP53-regulated inhibitor of apoptosis 1
WBC: White blood cell
